# Acute bacterial encephalitis complicated with recurrent nasopharyngeal carcinoma associated with *Elizabethkingia miricola* infection: A case report

**DOI:** 10.3389/fneur.2022.965939

**Published:** 2023-01-27

**Authors:** Xiaohuang Zhuo, Yongzhao Zhou, Ling Liu

**Affiliations:** ^1^Department of Neurology, West China Hospital, Sichuan University, Chengdu, China; ^2^Precision Medicine Key Laboratory of Sichuan Province and Precision Medicine Center, West China Hospital, Sichuan University, Chengdu, China

**Keywords:** *Elizabethkingia miricola*, encephalitis, metagenomic next-generation sequencing, diagnosis, treatment

## Abstract

*Elizabethkingia miricola* (*E. miricola*) is an extremely rare pathogenic bacterium, which causes serious infections in patients with primary immunodeficiency or tumors, and it is often misdiagnosed. *E. miricola* has rarely been known to cause a neurologic infection. We describe the first case of acute bacterial encephalitis associated with *E. miricola* infection in a man with recurrent nasopharyngeal carcinoma, which was successfully cured by antibiotics. The patient initially presented with recurrent episodes of fever and later showed impaired consciousness but these symptoms were alleviated with antibiotic therapy including cefoperazone/sulbactam. This study highlights that rapid and accurate pathogen detection *via* metagenomic next-generation sequencing and early use of appropriate antibiotics can improve the prognosis of patients with suspected neurologic *E. miricola* infection. Early treatment for underlying primary diseases can also significantly improve the outcomes of patients.

## Introduction

*Elizabethkingia miricola* (*E. miricola*) is a non-fermenting Gram-negative bacterium that was first discovered in 2003 when it was isolated from condensation water in the Russian Space Laboratory Mir (1). Generally, *E. miricola* does not cause infections in healthy populations, but it is a serious conditional pathogen affecting individuals with compromised immunity. For instance, *E. miricola* was found to cause infection in an allogeneic stem cell transplant recipient with mantle cell lymphoma (2). It has also been reported to cause urinary tract infection in a female with abdominal pain (3), knee septic arthritis in a male patient with recurrent erysipelas (4), and oral superinfection in a woman with common variable immunodeficiency (5). However, *E. miricola* is rarely known to cause a neurologic infection. To date, only one case of meningoencephalitis caused by *E. miricola* has been reported, and the patient eventually died due to symptoms of aggravation (6). In addition, medically important species of *Elizabethkingia* include *Elizabethkingia meningosepticum, Elizabethkingia anophelis*, and *E. miricola*. The currently used routine morphological, biochemical, and molecular tests cannot accurately distinguish *E. miricola* from other *Elizabethkingia* species. Previous studies have also demonstrated that *E. miricola* was frequently misidentified as *E. meningosepticum* initially (2, 6). Given the limited reports on the diagnosis and treatment of this infection, the neurologic infections caused by *E. miricola* are poorly understood. The aim of our study was to describe another case of bacterial encephalitis caused by *E. miricola* that was diagnosed early and accurately and treated successfully.

## Case report

A 56-year-old man presented with recurrent episodes of fever with no trigger for 3 weeks and disturbance of consciousness for 2 weeks. The patient had undergone chemo-radiation treatment for nasopharyngeal carcinoma (NPC) 18 years ago. He denied a history of traumatic brain injury. The travel history of the patient was unremarkable. Neurologic examination revealed somnolence, confusion, enlargement of the left pupil with absent reaction to light, hearing decline in the left ear, normal muscle strength and tension in limbs, bilateral positive Chaddock signs, negative meningeal irritation signs, and Babinski signs.

**Figure 1:**
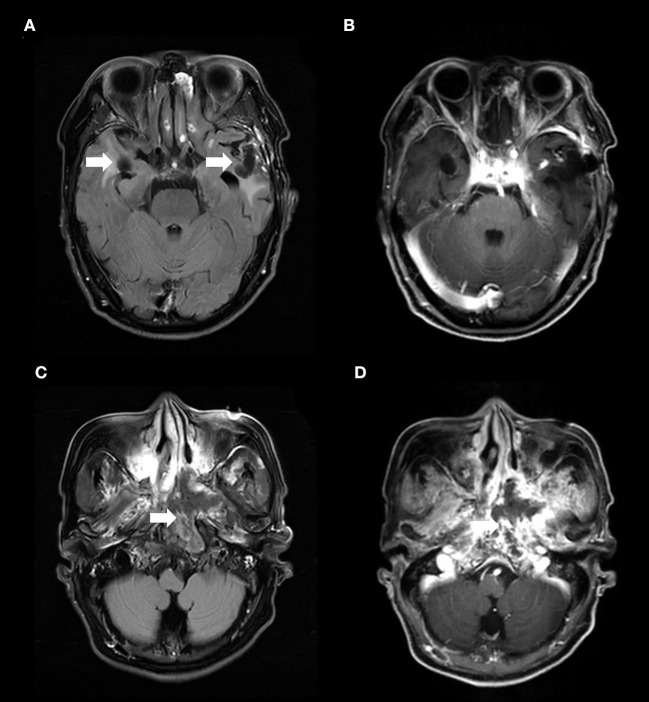
**(A)** The axial T2-weighted FLAIR imaging demonstrated irregular hypointense signal changes with patchy edema around them in the bilateral temporal lobes (arrows) and **(B)** without contrast enhancement in the similar brain structures. The MRI abnormalities on bilateral temporal lobes had been present for approximately 5 months before the patient's disturbance of consciousness. These abnormalities were presentation of radiation-induced brain necrosis and were not associated with *E. miricola* encephalitis. **(C)** Nasopharynx T2-weighted FLAIR imaging showed a heterogeneous signal mass in the left nasopharyngeal lateral wall (arrow). **(D)** T1-weighted nasopharynx MRI with contrast enhancement showed enhancing lesions in the mass (arrow). FLAIR, fluid-attenuated inversion-recovery.

**Figure 2:**
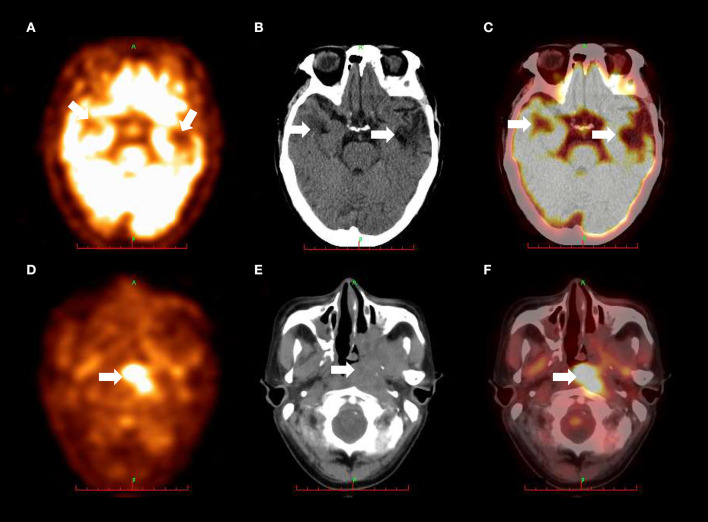
**(A)** PET scan revealed decreased *fluorodeoxyglucose* uptake in the bilateral temporal lobes (arrows). **(B)** CT scan showed patchy low-density areas in the bilateral temporal lobes (arrows). **(C)** ^18^F-FDG PET/CT fusion imaging demonstrated decreased *fluorodeoxyglucose* uptake in the bilateral temporal lobes (arrows). These metabolic abnormalities on bilateral temporal lobes were appearance of radiation-induced brain necrosis and were not correlated with *E. miricola* encephalitis. **(D)** PET scan revealed increased *fluorodeoxyglucose* uptake in the left nasopharyngeal lateral wall (arrow). **(E)** CT scan showed soft tissue mass with unclear boundary in the left nasopharyngeal lateral wall (arrow). **(F)** ^18^F-FDG PET/CT fusion imaging demonstrated increased *fluorodeoxyglucose* uptake in the left nasopharyngeal lateral wall (arrow). ^18^F-FDG PET/CT, and ^18^F-Fluoro-2-deoxyglucose positron emission tomography/computed tomography.

Magnetic resonance imaging (MRI) of the brain revealed irregular hypointense signal changes with patchy edema around them in the bilateral temporal lobes (Figure 1A), without enhancing lesions in the same brain structures (Figure 1B). Nasopharynx MRI showed a heterogeneous signal mass in the left nasopharyngeal lateral wall (Figure 1C) and enhancement in the mass (Figure 1D). ^18^F-Fluoro-2-deoxyglucose (^18^F-FDG) positron emission tomography/computed tomography (PET/CT) scan demonstrated decreased fluorodeoxyglucose uptake lesions in the bilateral temporal lobes (Figures 2A–C) and increased fluorodeoxyglucose uptake lesions in the left nasopharyngeal lateral wall (Figures 2D–F).

A lumbar puncture was performed, and empirical antimicrobial treatment with cefmetazole was started. The cerebrospinal fluid (CSF) was cloudy and had a leukocyte count of 18 × 10^6^ cells/L, a protein level of 3.60 g/L (normal: 0.15–0.45 g/L), a glucose level of 2.41 mmol/L (normal: 2.5–4.4 mmol/L), and a chloride level of 116 mmol/L (normal: 120–130 mmol/L). A culture of the CSF from the patient showed no growth for 5 days. Metagenomic next-generation sequencing (mNGS) of the CSF indicated *E. miricola* (sequence reads 115; MGISEQ-2000 platform, MGI Tech, China). The test results for immunity antibodies, parasites, other bacteria, and viruses were all negative. These results suggested a diagnosis of encephalitis caused by *E. miricola*.

The patient's treatment was then changed to intravenous cefoperazone/sulbactam (3 g, two times per day) after the diagnosis. Two weeks later, the patient's symptoms were remarkably alleviated. Reexamination of the CSF mNGS revealed that *E. miricola* was completely cleared and the follow-up CSF culture was sterile. A biopsy of the mass in the nasopharynx showed well-differentiated squamous cell carcinoma, which indicated recurrent NPC. Serological Epstein–Barr Virus DNA result was negative. The patient was transferred to the Department of Radiation Oncology and received chemo-radiation treatment for NPC, and he is presently in stable condition. The timeline of disease manifestations and corresponding treatment regimens are presented in Tables 1, 2, respectively.

**Table 1:** Timeline of disease manifestations.

**Disease manifestations**	**18 years prior**	**3 weeks prior**	**2 weeks prior**	**Day 0**	**Day 6**	**Day 15**	**Day 16-19**	**Day 20**	**8 months later**
Nasopharyngeal carcinoma									
Fever									
Disturbance of consciousness									
Somnolence									
Hearing decline									
Steady improvement									

**Table 2:** Timeline of treatment regimens.

**Treatment regimens**	**18 years prior**	**Day 0**	**Day 6**	**Day 11**	**Day 12**	**Day 19**	**Day 26**	**1 months later**	**2 months later**	**3 months later**
Chemo-radiation										
Cefmetazole (1.0 g q12 h)										
Aciclovir injection (0.5g q8 h)										
Cefoperazone/sulbactam (3.0 g q12h)										

## Discussion

In this report, we describe the first case of bacterial encephalitis associated with *E. miricola* infection that was successfully treated with antibiotics. *E. miricola* has been isolated from the blood, sputum, urine, and synovial fluid and has been found to cause sepsis, pneumonia, urinary tract infection, and knee septic arthritis (2–4, 7). However, *E. miricola* is rarely known to cause a neurologic infection in humans. Globally, only one case of meningoencephalitis caused by *E. miricola* has been reported, and the patient did not receive a timely diagnosis and treatment. Consequently, the patient died a few days after being discharged from the hospital (6). Due to the rarity and unknown etiology of the disease in the central nervous system, its diagnosis and treatment remain poorly understood.

The etiology of acute encephalitis cases is not identified in approximately 50% of patients (8). Failure to obtain a timely diagnosis in patients with central nervous system infections contributes to severe outcomes (9). *E. miricola* is an extremely rare pathogenic bacterium that is usually misidentified or considered to be other *Elizabethkingia* species or contaminants. This potentially masks the exact clinical significance of the bacterium. The first case of intracranial *E. miricola* infection was initially misdiagnosed as *E. meningosepticum*, which led to a delay in diagnosis and treatment (6). Therefore, it is challenging to determine the pathogenic role of infrequent isolates in patients with low immunity. Currently, the common detection methods for identifying *Elizabethkingia* include matrix-assisted laser desorption ionization-time of flight mass spectrometry, 16S rRNA gene, and mNGS (4, 10, 11). The former two methods are relatively inefficient and time-consuming since both of them require culturing of samples taken from sterile sites such as blood and CSF, which may not be applicable to patients with rapidly progressing infections. In comparison, mNGS is a one-step, culture-independent approach used for the detection of all pathogens from a single specimen. With the technological advancements of mNGS, the identification of *E. miricola* has been improved, and this has led to a better understanding of this uncommonly isolated microorganism (10). In this case, *E. miricola* was accurately identified as the causative pathogen by mNGS, and we considered this bacterium to be the main cause of encephalitis.

In addition, *E. miricola* is known to be multidrug-resistant, and there is no best-known therapy for neurologic *E. miricola* infection (6, 11). A previous study has revealed that *E. miricola* in neurologic infection was resistant to ceftriaxone and imipenem but susceptible to tigecycline, cefoperazone/sulbactam, levofloxacin, among other drugs (6). Similar to the previously reported case of *E. miricola* infection, our patient received cefoperazone/sulbactam therapy for 2 weeks during hospitalization, and his symptoms were significantly relieved. The reexamination by mNGS showed 0 *E. miricola* reads in the CSF. This suggested that cefoperazone/sulbactam effectively treated the neurologic *E. miricola* infection. Therefore, it can be used to treat similar patients with suspected neurologic *E. miricola* infection.

Most patients with bacteremia, sepsis, knee septic arthritis, and oral superinfection caused by *E. miricola* had underlying comorbidities, such as cancer and immunodeficiency (2, 4, 5). Furthermore, *Elizabethkingia* infections in patients with underlying diseases were usually associated with poor prognosis. Thus, therapeutic interventions for underlying primary diseases can remarkably prevent severe outcomes of *E. miricola* infection. A previous study has demonstrated that high-dose immunoglobulin and targeted levofloxacin treatment could result in immune system reconstitution, oral healing, and eradication of *Elizabethkingia* infection in a female diagnosed with common variable immunodeficiency (5). We also detected NPC recurrence in our patients who received timely chemo-radiation treatment. The 8-month follow-up indicated that the patient had a good prognosis. Therefore, in addition to the need for early identification of pathogens in patients with encephalitis, timely and extensive screening is necessary to determine whether patients have potential tumors or primary immunodeficiency.

Overall, this case study has two strengths. First, *E. miricola* infection was rapidly diagnosed using the unbiased mNGS, which proved to be more sensitive than conventional methods such as CSF smear and culture. Furthermore, the follow-up information of the patient was available, and this could help to evaluate the long-term prognosis of neurologic *E. miricola* infection. Nevertheless, a limitation is also present in this study, the association between cancers and neurologic *E. miricola* infection could not be identified, and further research is necessary to determine this relationship.

In conclusion, we report the first case of bacterial encephalitis after *E. miricola* infection that was cured by antibiotics. Our case may provide novel insights into the treatment of patients with *E. miricola* encephalitis. Rapid and accurate pathogen detection *via* mNGS and early use of appropriate antibiotics can improve the prognosis of patients with suspected neurologic *E. miricola* infection. Moreover, our case also extends the spectrum of pathogens known to cause encephalitis. Finally, early treatment of the underlying primary diseases can also significantly improve the outcomes of patients.

## Data availability statement

The raw data supporting the conclusions of this article will be made available by the authors, without undue reservation.

## Ethics statement

The studies involving human participants were reviewed and approved by Ethics Committee on Human Research of West China Hospital. The patients/participants provided their written informed consent to participate in this study.

## Author contributions

XZ wrote the draft of the manuscript, collected the clinical data, and designed the ideas of the article. YZ collected the clinical data. LL designed the ideas of the article and edited the whole manuscript. All authors contributed to the article and approved the submitted version.
